# Medical Association of Georgia

**Published:** 1888-05

**Authors:** 


### MEDICAL ASSOCIATION OF GEORGIA.

Our managing editor did himself the pleasure of attending the late meeting of the Medical Association of Georgia, which assembled at Rome on Wednesday at 12:30, the 18th of February, and continued in session until Friday at 3 p. m. Our space will permit only a brief account of the meeting.

The address of welcome, by Dr. Robt. Battey, was cordial, appropriate and well delivered.

Mr. John T. Graves, Editor of the Rome Tribune, at the request of Mayor Ayers, addressed the Association with additional words of welcome. His address was characterized by the spice, wit and eloquence for which editor Graves is noted. He is emphatically like cold souse, always ready for any occasion. He closed with the following cheering words: "And so, gentlemen, there is nothing for you to do but to enjoy yourselves. I recall that firm old chestnut of Marcelus Tullius Cicero—"Si bine vales, valeo," (If you are well I am well,) Everybody is well here and everybody is glad to see you, make yourselves at home. The city belongs to you and everybody echoes from the heart the sentiment " God bless the Doctors!"

Dr. Todd, of Atlanta, responded very happily in behalf of the profession. He complimented certain of the Doctors of the city, the city itself and its ladies.

President Whitehead's address followed. It consisted of a *Partial* sketch of the history of the Association from its early organization to the present time.

In the afternoon session of the first day Reports of Sections was called for, but we regret to say that as usual very few responded.

The present anatomical law was referred to and commended as having furnished about 26 subjects for dissection, the first year of its trial. It was said by a member that the entire credit for the passage of this law was due to the late Dr. Jas. A Gray. No credit was given to the editors of this journal, through whose agency certain obnoxious features in the original bill were stricken or modified.

There were a number of voluntary papers presented and a limited number of reports, but a very few read or discussed. Want of time seemed to be the difficulty, due we think in a great measure to the fact that the order of business was not adhered to and parliamentary law not sufficiently observed.

The Treasurer made a good report showing a balance in the treasury of $487

The Committee on Prize Essays reported $100 in hand.

We did not procure the names of all who handed in or read papers, but recall the following: Drs. Todd, Moore, Medlock, Calhoun, Hardon, Word, Cortilyou, Cooper, T. M. Holmes, Dekle, Cotter, Davidson, Gaston, Taylor and Noble.

The Committee on Necrology reported deaths during the year of Dr. James A. Gray, of Atlanta, Dr. D. W. Hammond, of Macon, Dr. T. F. Walker, of Cochran, Ga , V. H. Taliaferro, of Atlanta, L. E. Borcheim, Atlanta, and J. J. Warring, of Savannah.

Dr. Jno. Thad. Johnson, a member once prominent in the Association, but who had been absent from the State on account of sickness, appeared upon the floor and was by unanimous vote elected an honorary member of the body.

Invitations were received from both Brunswick and Macon to meet there next time. After a short discussion it was decided by ballot to hold the 40th annual sesssion in Macon, beginning the third Wednesday in April, 1889.

The Nominating Committee reported the following officers for the ensuing year:

President, J. S. Todd, Atlanta.
First Vice President, J. B. S. Holmes, Rome.
Second Vice President, E. R. Anthony, Griffin.
Secretary, K. P. Moore, Macon.
Censor, B. R. Doster, Blakely.

The president announced the following appointments of committees and orator

Committee on Publication.—K. P. Moore, E. C. Goodrich, T. O. Powell, H. McHattan, W. F. Holt, Wm. O'Daniel.

Committee on Necrology.—Eugene Foster, G. W. Holmes, H. P. Cooper, R. J. Nunn, M. H. O'Daniel.

Committee on Prize Essays —R. J. Nunn, W. A. Love, H. F. Campbell, Robert Battey.

Committee on Arrangements.—W. F. Holt, C. H Hall, W. R. Winchester, H. J. Williams, H. McHatton.

Orator.—T. M. Holmes.

The president announced the following delegates to the American Medical Association: J. S. Todd, Robert Battey, Wm. O'Daniel, T. O Powell, T. S. Powell, Eugene Foster, K. P. Moore, T. M. McIntosh, R. C. Word, J. B S. Holmes, H B. McMaster, W. F. Westmoreland, A. W. Calhoun, E. H. Richardson, R. J. Nunn, E. C. Goodrich, H. McHatton, W. F. Westmoreland, Jr., J. H. Goss, T. S. Dekle, C. H. Colding, P. L. Cortelyou, G. W. Mulligan, B. R. Doster, J. W. Bailey, W. H. Doughty, Jr, W. A. Love, W. B. Wells, R. B. Barron, R. H. Taylor.

The following resolution was adopted: *Be it resolved*, first, That a committee on programme be appointed annually by the association, consisting of seven members, who shall have power to formulate the programme for the workings of the Association in its meetings, and in so doing shall co-operate with the local committee.

Second. That the title to all papers to be read by members of the association shall be sent to the chairman of the committee, at least thirty days before the meeting of the association, and that this committee appoint and notify the members who shall lead in the discussion of such papers and shall notify all members of the association as to the programme to be followed.

Third. That this resolution shall not in any way or manner abridge the right of any member to read a voluntary paper or to take part in these discussions.

Dr. Hardon, of Atlanta, presented the following names for the committee on programme. Upon motion they were duly appointed.

H. P. Cooper, S. C. Benedict, T. M. Holmes, J. J. Hill, W. H. Doughty, Jr., W. F. Westmoreland, K. P. Moore.

Dr. Benedict offered a resolution changing the present method of selecting district committees to report on special subjects. Dr. A. W. Calhoun offered as an amendment that the number of the district committee be ten, one from each congressional district. The amended resolution passed as follows:

*Be it resolved*, That the power of the nominating committee shal. be restricted to the nomination of officers of the association, and that a special committee consisting of one member from each congressional district shall be appointed by the secretary, whose duties shall be to name the members in each district to report upon the special subjects for each succeeding meeting, and that the committee be known as the Committee of Districts.

Dr. Benedict moved the adoption of the following resolution and it was carried unanimously:

*Be it resolved*, That the committee on reorganization of the association be discontinued, and that reports on Necrology be no longer read before the association, but published in the transactions as provided by the by-laws.

We have not the space to detail the particulars of the various entertainments, excursions, etc., extended by the profession and the citizens of Rome, to the members of the Association. Brilliant and enjoyable receptions were given by Drs. J. B. S. Holmes and Dr. Robt. Battey. Splendid concerts by the Rome Female College, and the Shorter College. An excursion on the dummy line to Lake View, and a barbecue. Excursion on steam boat down the Coosa river, etc.

Free travel upon street cars was given the members during their stay, and the citizens generally extended a warm greeting and a hospitable hand to the visiting doctors.

